# Generation of Highly Functional Hepatocyte-like Organoids from Human Adipose-Derived Mesenchymal Stem Cells Cultured with Endothelial Cells

**DOI:** 10.3390/cells13060547

**Published:** 2024-03-20

**Authors:** Shuhai Chen, Yu Saito, Yuhei Waki, Tetsuya Ikemoto, Hiroki Teraoku, Shinichiro Yamada, Yuji Morine, Mitsuo Shimada

**Affiliations:** Department of Surgery, Tokushima University, 3-18-15 Kuramoto-cho, Tokushima 770-8503, Japan; chen18354276982@163.com (S.C.); esperanza0813@gmail.com (Y.W.); ikemoto.tetsuya@tokushima-u.ac.jp (T.I.); teraoku.hiroki@tokushima-u.ac.jp (H.T.); yamada.shinichirou@tokushima-u.ac.jp (S.Y.); ymorine@tokushima-u.ac.jp (Y.M.); ichigeka@tokushima-u.ac.jp (M.S.)

**Keywords:** hepatocyte-like organoids, three-dimensional culture, mesenchymal stem cells, endothelial cells, immunogenicity properties

## Abstract

Previously, we successfully established a highly functional, three-dimensional hepatocyte-like cell (3D-HLC) model from adipose-derived mesenchymal stem cells (ADSCs) via a three-step differentiation protocol. The aim of the present study was to investigate whether generating hepatocyte-like organoids (H-organoids) by adding endothelial cells further improved the liver-like functionality of 3D-HLCs and to assess H-organoids’ immunogenicity properties. Genes representing liver maturation and function were detected by quantitative reverse transcription–PCR analysis. The expression of hepatic maturation proteins was measured using immunofluorescence staining. Cytochrome P (CYP)450 metabolism activity and ammonia metabolism tests were used to assess liver function. H-organoids were successfully established by adding human umbilical vein endothelial cells at the beginning of the definitive endoderm stage in our 3D differentiation protocol. The gene expression of alpha-1 antitrypsin, carbamoyl–phosphate synthase 1, and apolipoprotein E, which represent liver maturation state and function, was higher in H-organoids than non-organoid 3D-HLCs. H-organoids possessed higher CYP3A4 metabolism activity and comparable ammonia metabolism capacity than 3D-HLCs. Moreover, although H-organoids expressed human leukocyte antigen class I, they expressed little human leukocyte antigen class II, cluster of differentiation (CD)40, CD80, CD86, and programmed cell death ligand 1, suggesting their immunogenicity properties were not significantly upregulated during differentiation from ADSCs. In conclusion, we successfully established an H-organoid model with higher liver-like functionality than previously established 3D-HLCs and comparable immunogenicity to ADSCs.

## 1. Introduction

A wide range of both acute and chronic liver injuries can evolve into irreversible liver failure, which has become a global health burden, causing approximately 2 million deaths each year [1]. Whole-organ liver transplantation is performed worldwide as a standard treatment for severe liver disease; however, donor shortages and immune rejection problems limit its clinical application [2]. Hepatocyte transplantation is a viable alternative to liver transplantation and has been used as a bridging treatment for patients with metabolic disorder syndrome or acute liver failure [3]. In our previous study, we successfully established three-dimensionally cultured hepatocyte-like cells (3D-HLCs) from adipose-derived mesenchymal stem cells (ADSCs) using human recombinant peptide μ-pieces as scaffolds. These 3D-HLCs present an alternative source of primary hepatocytes with great potential for use in regenerative medicine [4]. However, high-quality, differentiated cells with more physiological liver functions are crucial to increase the value of 3D-HLCs in clinical applications. Moreover, the immune characteristics of these stem cell-derived cell products still need further evaluation.

In recent years, organoid cell culture technology has developed rapidly [5]. These multicellular systems can provide suitable microenvironments for the differentiation of stem cells [6]. Specifically, the use of endothelial cells in hepatic lineage differentiation of stem cells is well established; however, these models mostly use induced pluripotent stem cells, and their immunological properties have not been evaluated [7].

Therefore, in the present study, we added endothelial cells during our previously established 3D-HLC differentiation protocol to generate H-organoids and investigate their functionality and immune properties. We successfully generated H-organoids that had better liver-like functions than previous, non-organoid 3D-HLCs while maintaining lower immunogenicity similar to ADSCs.

## 2. Materials and Methods

### 2.1. ADSC Isolation

A 78-year-old male donor who had undergone laparoscopic cholecystectomy at Tokushima University in 2021 was included in this study. Subcutaneous adipose tissue just below the umbilicus was collected during an operation. This study was authorized in advance by the Institutional Review Board of the Tokushima University Hospital (approved ID number: 3090) and the University Hospital Medical Information Network (approved ID number: 000035546) and was performed in accordance with the relevant guidelines and regulations. The patient provided written informed consent.

ADSCs were isolated from adipose tissue using a commercial ADSC isolation kit (Funakoshi Co., Ltd., Tokyo, Japan) as described previously [8]. Briefly, diced adipose tissue was cultured in a 3D, hydroxyapatite-coated, polyethylene–polypropylene, nonwoven fabric matrix for 2 weeks. Isolated ADSCs were then collected by trypsinization and cultured in PRIME-XV MSC XSFM MDF1 medium (552-37463, FUJIFILM, Tokyo, Japan) supplemented with 10% NeoSERA (JBM001, FUJIFILM). Human umbilical vein endothelial cells (HUVECs, C2519A) were purchased from LONZA and cultured in Endothelial Cell Growth Medium-2 (CC-3162, LONZA, Basel, Switzerland) at 90% confluence until use.

### 2.2. HLC and H-Organoid Differentiation

ADSCs were differentiated into HLCs in 2D and 3D patterns according to our previously established three-step protocol and named 2D-HLCs and 3D-HLCs, respectively [4]. In brief, ADSCs were seeded into collagen I-coated six-well plates (4810-010; IWAKI, Tokyo, Japan; 2 × 10^5^ cells per well for the 2D culture pattern) or ultra-low attachment 96-well plates (174925; Thermo Fisher Scientific, Waltham, MA, USA; 2 × 10^4^ cells mixed with 0.02 mg human recombinant peptide μ-pieces from FUJIFILM per well for the 3D culture pattern). The μ-pieces act as scaffolds for ADSCs. The features of this RCP include that it is xeno-antigen free and has high cell adhesiveness, biodegradation absorption, and stable manufacturing quality [4]. After this, a three-step sequential cocktail medium containing only xenobiotics was used to induce hepatocyte differentiation, as previously reported [4]. For the addition of HUVECs into 3D-HLCs at different steps, HUVECs were detached by trypsin and added to HLCs at a ratio of 1:3 cells.

### 2.3. Morphological Analysis

The morphology of 3D-HLCs and 3D-HLCs with HUVECs was observed and photographed by a light microscope (magnification, ×100; DP22-CU; Olympus, Tokyo, Japan). For hematoxylin–eosin staining, indicated spheres were solidified using iPGell (PG20-1; Genostaff, Osaka, Japan) and fixed in 10% paraformaldehyde, as previously described [9]. Next, 4 μm thick paraffin-embedded sections were prepared, and hematoxylin–eosin staining was performed according to a standard protocol.

### 2.4. Quantitative Reverse Transcription–PCR (qRT-PCR) Analysis

An RNeasy Mini Kit (Qiagen, Hilden, Germany) was used to extract total RNA from each sample; cDNA was then synthesized using a reverse transcription kit (Applied Biosystems, Thermo Fisher Scientific Inc., Tokyo, Japan). The StepOnePlus Real-Time PCR System (Applied Biosystems, Thermo Fisher Scientific Inc.) was used to perform TaqMan-qRT-PCR analyses. The primers (TaqMan gene expression assays; Thermo Fisher Scientific Inc.) used were alpha-1 antitrypsin (*AAT*) (assay ID, Hs00165475_m1), α-fetoprotein (assay ID, Hs00173490_m1), albumin (*ALB*) (assay ID, Hs00609411_m1), carbamoyl-phosphate synthase 1 (*CPS1*) (assay ID, Hs00157048_m1), and apolipoprotein E (*APOE*) (assay ID, Hs00171168_m1). Glyceraldehyde 3-phosphate dehydrogenase (assay ID, 4326317E) was used as the internal control.

### 2.5. Fluorescence-Activated Cell Sorting (FACS) Analysis

Surface markers of ADSCs and differentiated H-organoids were analyzed using FACSVerse and BD FACSuite software v 1.0 (BD Biosciences, San Jose, CA, USA), as described previously [9]. To dissociate cells from μ-pieces, spheres were washed with phosphate-buffered saline (PBS) and incubated with 4 mg/mL collagenase type IV (17104-019, Thermo Fisher Scientific Inc.) in Hank’s balanced salt solution (HBSS) for 1 h at 37 °C. The antibodies used were as follows: human leukocyte antigen (HLA)-class I (Catalog # 14-9983-82, PE-conjugated; e-Bioscience, San Diego, CA, USA), HLA class II (Catalog # 12-9956-42, PE-conjugated; BioLegend, San Diego, CA, USA), cluster of differentiation (CD)31 (Catalog # 14-0311-82, PE-conjugated; e-Bioscience), CD40 (Catalog #334302, PE-conjugated; BioLegend), CD45 (Catalog #304007, PerCP/Cy5.5-conjugated; BioLegend), CD80 (Catalog #305201, PE-conjugated; BioLegend), CD86 (Catalog #374205, PE-conjugated; BioLegend), CD90 (Catalog # 14-0909-82, APC-conjugated; e-Bioscience), CD105 (Catalog # 14-1057-82, APC-conjugated; e-Bioscience), and programmed cell death ligand 1 (PD-L1) (Catalog #329705, PE-conjugated; BioLegend).

### 2.6. Live/Dead Cell Staining

The LIVE/DEAD Cell Imaging Kit (Cat. No. R37601, Thermo Fisher) was used to stain live and dead cells and visualize them as green or red via fluorescence microscopy. According to the manufacturer’s instructions, indicated cells were incubated in a 2× mixture of solution A and solution B at 20 °C for 15 min and then observed under a fluorescence microscope (BZ-X700; KEYENCE, Tokyo, Japan). All experiments were performed in at least three independent biological replicates, and three fields of view per well were randomly selected for quantitative analysis.

### 2.7. Immunofluorescence Staining

Both 3D-HLCs and H-organoids were transferred into a 10 mL tube, washed twice with PBS, and fixed with 4% paraformaldehyde (163-20145; FUJIFILM) at 4 °C for 1 h. After permeabilization with 0.1% Triton X-100 (HFH10; Thermo Fisher Scientific) for 10 min at room temperature, all samples were incubated with 3% bovine serum albumin overnight at 4 °C. Samples were then incubated with an anti-ALB antibody (1:200, 16475-1-AP, Proteintech, Tokyo, Japan) or an anti-hepatocyte nuclear factor 4 alpha (HNF4α) antibody (1:1000, 3113 s, Cell Signaling Technology, Inc., Tokyo, Japan) for 24 h at 4 °C. The next day, samples were thoroughly washed three times with PBS and incubated with Alexa Fluor 488-conjugated secondary antibodies (1:1000, A-11008, Thermo Fisher Scientific, Inc.) for 1 h at 37 °C in the dark. Next, samples were thoroughly washed again and transferred into a 24-well dish. Nuclei were stained for 10 min with ProLong Gold Antifade Mountant with DAPI (P36931; Thermo Fisher Scientific) before being observed and photographed under a fluorescence microscope. For negative controls, the primary antibody was replaced with bovine serum albumin.

### 2.8. CYP3A4 Activity Assay

CYP3A4 enzyme activity was assessed using a P450-Glo CYP3A4 Assay (V8801, Promega, Tokyo, Japan) in accordance with the manufacturer’s instructions and as previously reported [4]. Luminescence was measured with a luminometer (SpectraMax i3; Molecular Devices, LLC, CA, USA). CYP3A4 activity was expressed relative to cell number quantified by luminescence.

### 2.9. Ammonium Metabolism Assay

Ammonium metabolism was evaluated by the change in ammonium concentration in the cell culture supernatant 24 h after the addition of ammonium chloride (NH_4_Cl). Briefly, a standard of 300 µmol/L NH_4_Cl (FUJIFILM Wako Pure Chemical Corporation) diluted with HBSS (FUJIFILM Wako Pure Chemical Corporation) was added to the culture plates after the plates had been washed twice with HBSS. After this, the supernatants were collected, and ammonium concentrations were measured at 24 h intervals after NH_4_Cl addition using an ammonia assay kit (Cell, Biolabs, San Diego, CA, USA). Culture plates containing only the standard ammonia solution were used as controls.

### 2.10. Statistical Analysis

All data are presented as mean ± standard mean difference. Statistical analysis and graphing were conducted using GraphPad Prism v7.0 software (GraphPad Software, Inc., Boston, MA, USA) and Image J software v 1.53. Comparisons between two groups were analyzed using the unpaired Student’s *t*-test or the Mann–Whitney test. Differences among multiple groups were analyzed using a one-way ANOVA followed by Tukey’s post hoc tests. All experiments were repeated more than three times. *p*-values less than 0.05 (two-sided) were considered statistically significant.

## 3. Results

*3.1. 3D-HLCs with HUVECs*

**Figure 1:**
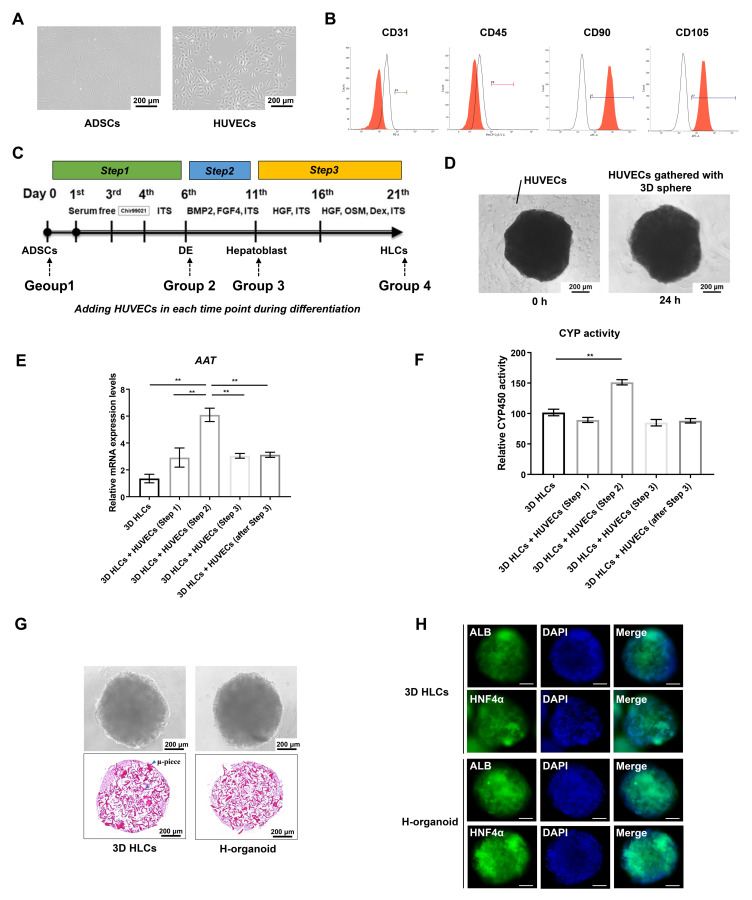
The establishment of H-organoids by adding HUVECs at the definitive endoderm stage of our 3D-HLC differentiation protocol. (**A**) Representative images showing the morphology of ADSCs and HUVECs under a light microscope. (**B**) FACS analysis showing the expression of surface markers CD31, CD45, CD90, and CD105 on isolated ADSCs. (**C**) Schematic diagram of the procedure for adding HUVECs at different time points during differentiation based on our 3D-HLC differentiation protocol. (**D**) Representative images of spheroids 0 h and 24 h after adding HUVECs to 3D-HLCs. (**E**) Relative gene expressions of *AAT* in 3D-HLCs with HUVECs added at different steps were evaluated by qRT-PCR. (**F**) Relative CYP3A4 activity in 3D-HLCs with HUVECs added at different steps. (**G**) Representative images of 3D-HLCs and H-organoids under a light microscope after HE staining. (**H**) Immunofluorescence staining showing the expression of ALB and HNF4α in 3D-HLCs and H-organoids. AAT: alpha-1 antitrypsin; ADSCs: adipose-derived mesenchymal stem cells; ALB: albumin; FACS: fluorescence-activated cell sorting; HE: hematoxylin–eosin; HNF4α: hepatocyte nuclear factor 4 alpha; H-organoids: hepatocyte-like organoids; HUVECs: human umbilical vein endothelial cells; qRT-PCR: quantitative reverse transcription–PCR; 3D-HLCs: three-dimensionally cultured hepatocyte-like cells; ** *p* < 0.01. Differences among multiple groups were analyzed using a one-way ANOVA followed by Tukey’s post hoc tests.

ADSCs isolated from the patient’s subcutaneous adipose tissue were passaged two to three times before use; morphologically, they were spindle-shaped (Figure 1A). FACS analysis confirmed that isolated and passaged ADSCs were positive for the expression of the mesenchymal markers CD90 and CD105 and negative for the expression of CD31 and CD45 (Figure 1B). The morphology of HUVECs under light microscopy was a typical “paving stone pattern” (Figure 1A). As shown in Figure 1C, four groups were established according to which step of differentiation HUVECs were added: Group 1, at the beginning of step 1; Group 2, at the beginning of step 2; Group 3, at the beginning of step 3; and Group 4, at the end of step 3. Figure 1D shows that the freshly added HUVECs were distributed around the spheroids, and after 24 h, HUVECs were gathered with the spheroids. Morphologically, well-formed spheroids were observed in all groups (Figure S1A), and each group had spheroids with a similar diameter (Figure S1B). However, *AAT*, a typical gene representing liver maturation, was most highly expressed in the group with the addition of HUVECs at the beginning of step 2 (Group 2), which is the definitive endoderm stage (Figure 1E). Moreover, CYP3A4 metabolic activity was also significantly higher in this group than in the other groups (Figure 1F). Therefore, we named the spheroids from this group of H-organoids and selected this model for the following experiments. The typical morphology of 3D-HLCs and H-organoids is presented in Figure 1G, with adequate HLC formation maintained by the μ-piece scaffolds. Figure 1H shows that typical markers of hepatocyte differentiation, ALB and HNF4α, were significantly expressed in both 3D-HLCs and H-organoids, suggesting efficient differentiation into the hepatic lineage.

### 3.2. H-Organoids Present with Better Liver-like Functions In Vitro

**Figure 2:**
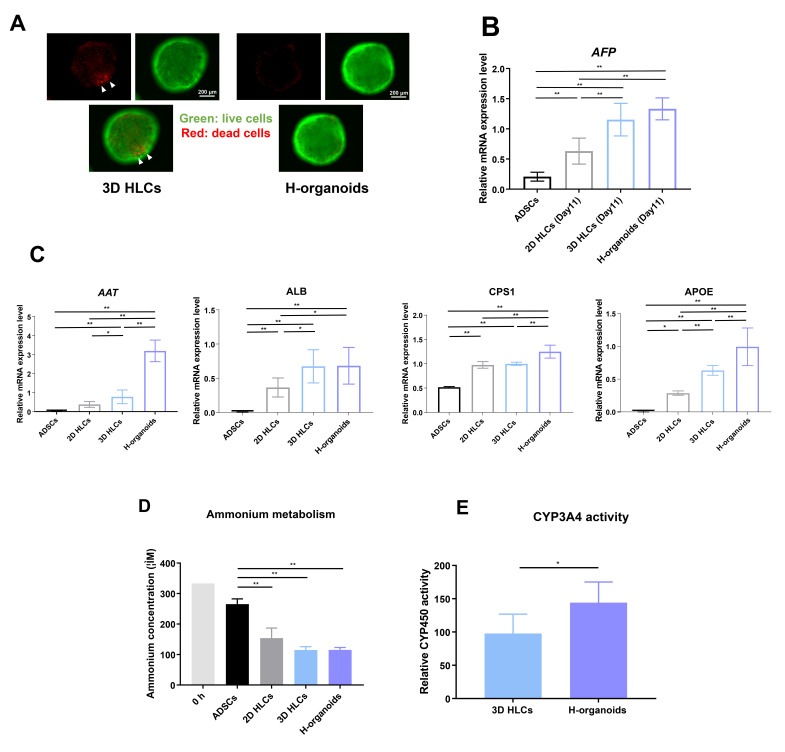
H-organoids present with better liver functions than 3D-HLCs in vitro. (**A**) Representative images showing live/dead cells in 3D-HLCs and H-organoids under a fluorescence microscope. (**B**) Relative expression of *AFP* in ADSCs and day 11-differentiated hepatic lineage cells evaluated by qRT-PCR. (**C**) Relative gene expression of *AAT*, *ALB*, *CPS1*, and *APOE* in ADSCs, 2D-HLCs, 3D-HLCs, and H-organoids. (**D**) Ammonium metabolism capacity in ADSCs, 2D-HLCs, 3D-HLCs, and H-organoids as measured by the residual ammonia concentration in the culture medium. (**E**) Relative CYP3A4 activity in 3D-HLCs and H-organoids. AAT: alpha-1 antitrypsin; ADSCs: adipose-derived mesenchymal stem cells; AFP: α-fetoprotein; ALB: albumin; APOE: apolipoprotein E; CPS1: carbamoyl–phosphate synthase 1; H-organoids: hepatocyte-like organoids; qRT-PCR: quantitative reverse transcription–PCR; 2D-HLCs: two-dimensionally cultured hepatocyte-like cells; 3D-HLCs: three-dimensionally cultured hepatocyte-like cells; * *p* < 0.05; ** *p* < 0.01. Comparisons between two groups were analyzed using the Mann–Whitney test. Differences among multiple groups were analyzed using a one-way ANOVA followed by Tukey’s post hoc tests.

Usually, the central necrosis of 3D spheres is an important issue that affects differentiation quality in spheroids. In our previously established protocol using μ-piece scaffolds, the diameter of spheroids was controlled to be within 400–800 μm and displayed little central necrosis (Figure 2A). However, compared with 3D-HLCs, H-organoids with added HUVECs had almost no central necrosis, predicting better cell viability. Next, we examined marker genes during differentiation into the hepatic lineage. At the hepatoblast stage, H-organoids tended to have higher α-fetoprotein than 3D-HLCs at the same stage (Figure 2B). The expression of *AAT*, *CPS1*, and *APOE*, genes that represent liver maturation state and function, were significantly higher in H-organoids than 3D-HLCs, with *ALB* expression comparably high in both H-organoids and 3D-HLCs (Figure 2C). Next, we performed liver function tests in vitro: results showed that H-organoids had a comparable advantage to 3D-HLCs in terms of ammonia metabolism (Figure 2D), while H-organoids performed better than 3D-HLCs in terms of CYP3A4 metabolism (Figure 2E).

### 3.3. Immunogenicity of H-Organoids during Differentiation from ADSCs

**Figure 3:**
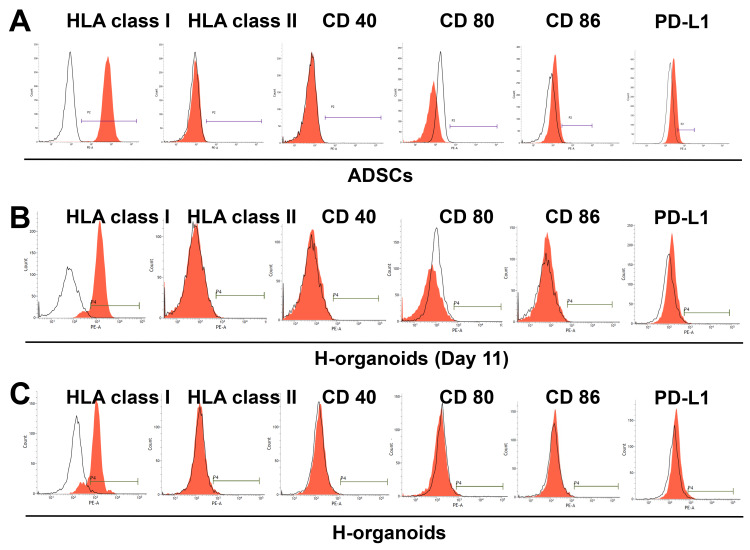
Expression of immune-related surface markers on ADSCs, day 11-differentiated H-organoids, and completely differentiated H-organoids. Expression of HLA class I, HLA class II, CD40, CD80, CD86, and PD-L1 on ADSCs (**A**), H-organoids (day 11) (**B**), and completely differentiated H-organoids (**C**) analyzed by FACS. ADSCs: adipose-derived mesenchymal stem cells; CD: cluster of differentiation; FACS: fluorescence-activated cell sorting; HLA: human leukocyte antigen; H-organoids: hepatocyte-like organoids; PD-L1: programmed cell death ligand 1.

Immunogenicity is another important issue for mesenchymal stem cell (MSC)-derived products when transplanted into patients in a clinical setting. To evaluate the immunogenicity properties of H-organoids during differentiation from ADSCs, we performed FACS analysis in ADSCs, in day 11-differentiated cells, and in completely differentiated H-organoids. ADSCs expressed HLA class I but not HLA class II CD40, and CD80; ADSCs had very weak expression of CD86 and PD-L1 (Figure 3A). Day 11-differentiated cells and completely differentiated H-organoids also expressed HLA class I, weakly expressed PD-L1, and barely expressed HLA class II, CD40, CD80, and CD86 (Figure 3B,C). These results indicate that H-organoids were not significantly more immunogenic than ADSCs.

In the present study, we successfully generated H-organoids from ADSCs by adding HUVECs at the beginning of step 2 of our established 3D-HLC differentiation protocol. Compared with previous 3D-HLCs, our newly generated H-organoids had higher liver-like functions but maintained lower immunogenicity similar to ADSCs; therefore, H-organoids appear to be a promising potential source for hepatocyte transplantation therapies in the future.

Currently, HLCs derived from stem cells are an important alternative source of primary hepatocytes in the field of regenerative medicine, but their differentiation maturity is still unsatisfactory [3]. Providing a multiple-cell microenvironment that mimics in situ development is an effective means to promote the differentiation of stem cells in vitro [6]. The outstanding study by Takebe et al. suggests that endothelial cells can help to generate vascularized and functional liver buds [10,11]. Endothelial cells are among the first cells to appear in the initial stages of embryogenesis, and the related signaling pathway is extremely important in liver organogenesis [11]. As our results show, H-organoids generated by adding HUVECs to ADSCs at the definitive endoderm stage of hepatic lineage differentiation resulted in a significant increase in expression of genes related to liver maturation and function, especially *AAT*, *CPS1*, and *APOE*. Moreover, H-organoid CYP metabolism activity was also enhanced. A previous study showed that endothelial cells can regulate α5β1 integrin and Src signaling to enhance transcription of forkhead box A2/HNF4α/pregnane X receptor and promote the functional metabolic maturation of HLCs [12]. In addition, a report from Liu et al. indicates that glial cell line-derived neurotrophic factor secreted by HUVECs helps promote the maturation of human hepatocyte-derived liver progenitor-like cells and enhances their tight junctions through MET phosphorylation [13]. Furthermore, evidence suggests that endothelial cells can promote graft pre-vascularization and further improve graft survival, as well as improve post-transplant performance by recruiting host vessels and stimulating host endothelial cells to release endogenous factors in response to vascular secretory signals [7].

Our H-organoids have several unique advantages. In previous reports of the establishment of hepatocyte-like organoids, the addition of MSCs was indispensable [6,14,15] because culturing stem cells-derived HLCs with HUVECs alone does not lead to the formation of 3D liver buds [10,15]. However, the addition of MSCs during the differentiation process inevitably leads to high population heterogeneity in the differentiated products. Based on our established 3D culture system, we found that hepatic cells mixed with HUVECs can become self-assembled H-organoids with appropriate sphere diameter, as well as improved cell activity and avoidance of central necrosis compared with spheroids without HUVECs. Recently, microgravity culture environments have become a hot topic in organoid research [16], and we are conducting relevant studies in anticipation of combining rotating wall vessel culture apparatus to enable further functional enhancement of our newly generated H-organoids [17].

Another advantage of our H-organoids is that they present with low immunogenicity; previous studies mostly involved hepatocyte-like organoids derived from induced pluripotent stem cells, and their immunogenicity has not been explored. There is evidence that HLCs derived from MSCs retain the anti-inflammatory and pro-regenerative functions of MSCs in addition to developing hepatocyte functions [18,19,20]. To our knowledge, this is the first report about the generation of H-organoids from ADSCs and our data show that the immunogenicity of H-organoids is similar to ADSCs. It is possible that the immunomodulatory ability possessed by ADSCs might also be retained in H-organoids. In organ transplantation, recipient T cells trigger a strong immune response by recognizing MHC class I and class II molecules [21]. Similar to other stem cell-derived products, H-organoids express MHC-I molecules. However, in the study by Romano et al., although these cells expressed MHC class I molecules, CD8+ T cells were not activated, possibly due to the lack of immune co-recognition molecules [22]. In contrast to the increased immunogenicity reported with the differentiation of MSCs into cardiomyocytes, chondrocytes, and osteocytes [23,24,25], our H-organoids still lack the expression of the immune co-recognition molecules CD40, CD80, and CD86. This implies that they have the same low immunogenicity as ADSCs and may be commercialized for allogeneic use. Of course, further evaluation of the immunomodulatory capacity of H-organoids is still needed. In addition, because in vivo environments will be significantly different from in vitro environments, experiments in in vivo models should be carried out to further test the immune properties of these cells [22,26,27].

One limitation of this study is that only in vitro experiments were performed; therapeutic effects in animal models of human liver disease were not explored. Furthermore, H-organoid functionality was not compared to that of primary human hepatocytes. Our previous reports [4] revealed that cells from 3D-HLC spheroids were relatively similar to primary hepatocytes according to whole genomic DNA microarray analysis. Therefore, H-organoids may also have a similar genomic pattern to primary hepatocytes, although further examination is necessary to confirm this.

## 4. Conclusions

In conclusion, our H-organoids had higher liver-like functions than a previously established 3D-HLC model while maintaining immunogenicity similar to ADSCs.

## Data Availability

The data sets used and/or analyzed during this study are available from the corresponding author upon reasonable request.

## Supplementary Materials

The following supporting information can be downloaded at: https://www.mdpi.com/article/10.3390/cells13060547/s1, Figure S1: Adding HUVECs in different time points during our three-step HLCs differentiation protocol.

## Abbreviations

alpha-1 antitrypsin	AAT
adipose-derived mesenchymal stem cells	ADSCs
α-fetoprotein	AFP
apolipoprotein E	APOE
bovine serum albumin	BSA
carbamoyl-phosphate synthase 1	CPS1
cytochrome P	CYP
Hanks’ Balanced Salt Solution	HBSS
hematoxylin–eosin	HE
hepatocyte nuclear factor 4 alpha	HNF4α
hepatocyte-like cells	HLCs
hepatocyte-like organoids	H-organoids
human umbilical vein endothelial cells	HUVECs
human leukocyte antigen	HLA
human umbilical vein endothelial cells	HUVECs
immunofluorescence	IF
induced pluripotent stem cells	iPSCs
phosphate-buffered saline	PBS
reverse transcription–PCR	qRT-PCR
three-dimensional	3D
