# Supplementary material for: Generation of Highly Functional Hepatocyte-like Organoids from Human Adipose-Derived Mesenchymal Stem Cells Cultured with Endothelial Cells
Source: Cells. 2024 Mar 20;13(6):547. doi: 10.3390/cells13060547 (PMC10969286; doi:10.3390/cells13060547)
Supplement: Supplementary file 1 [file cells-13-00547-s001.zip › cells-2868787-supplementary.pdf]

A

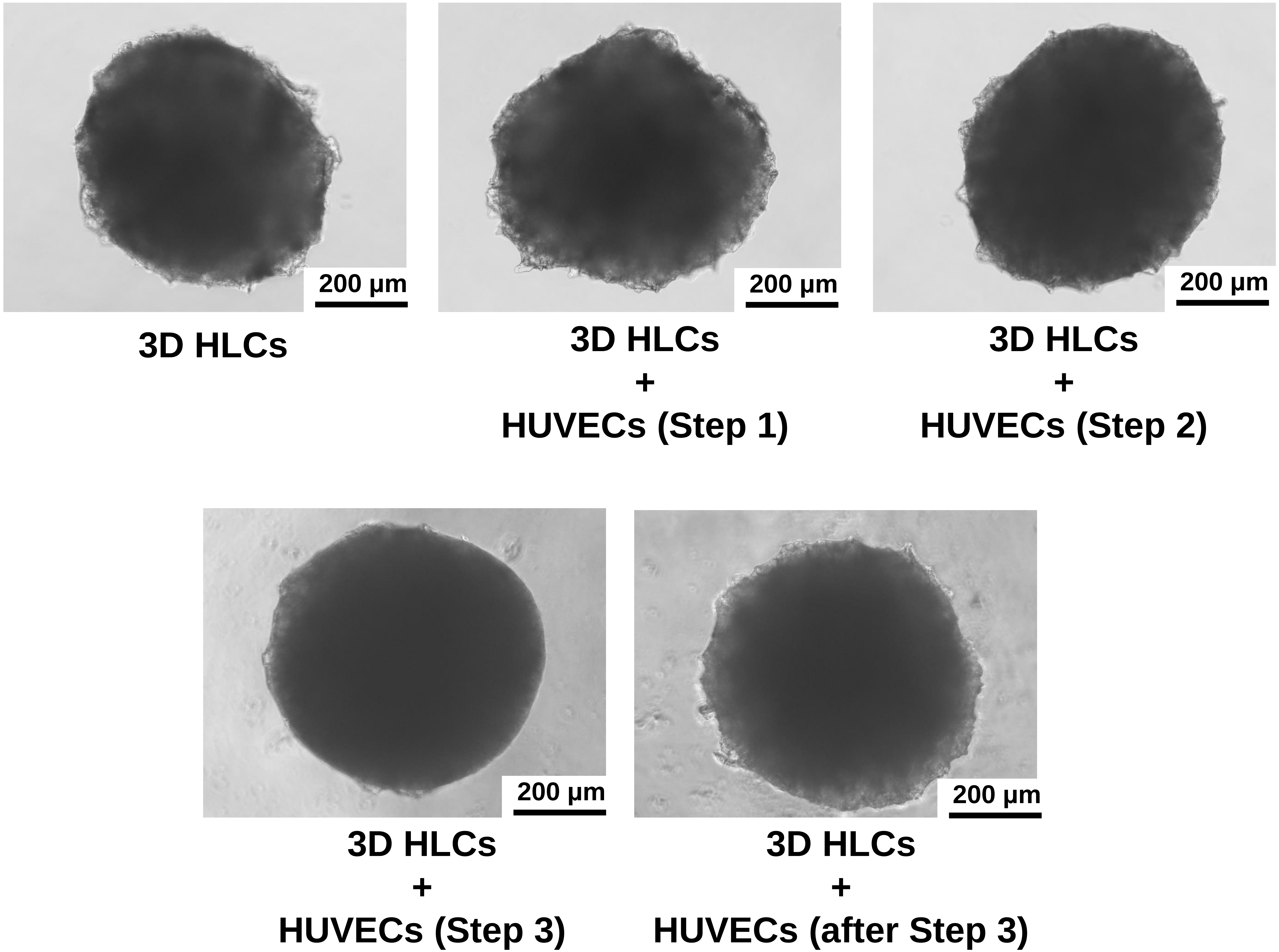
200 µm
3D HLCs
200 µm
3D HLCs
+
HUVECs (Step 1)
200 µm
3D HLCs
+
HUVECs (Step 2)
200 µm
3D HLCs
+
HUVECs (Step 3)
200 µm
3D HLCs
+
HUVECs (after Step 3)

S Figure 1

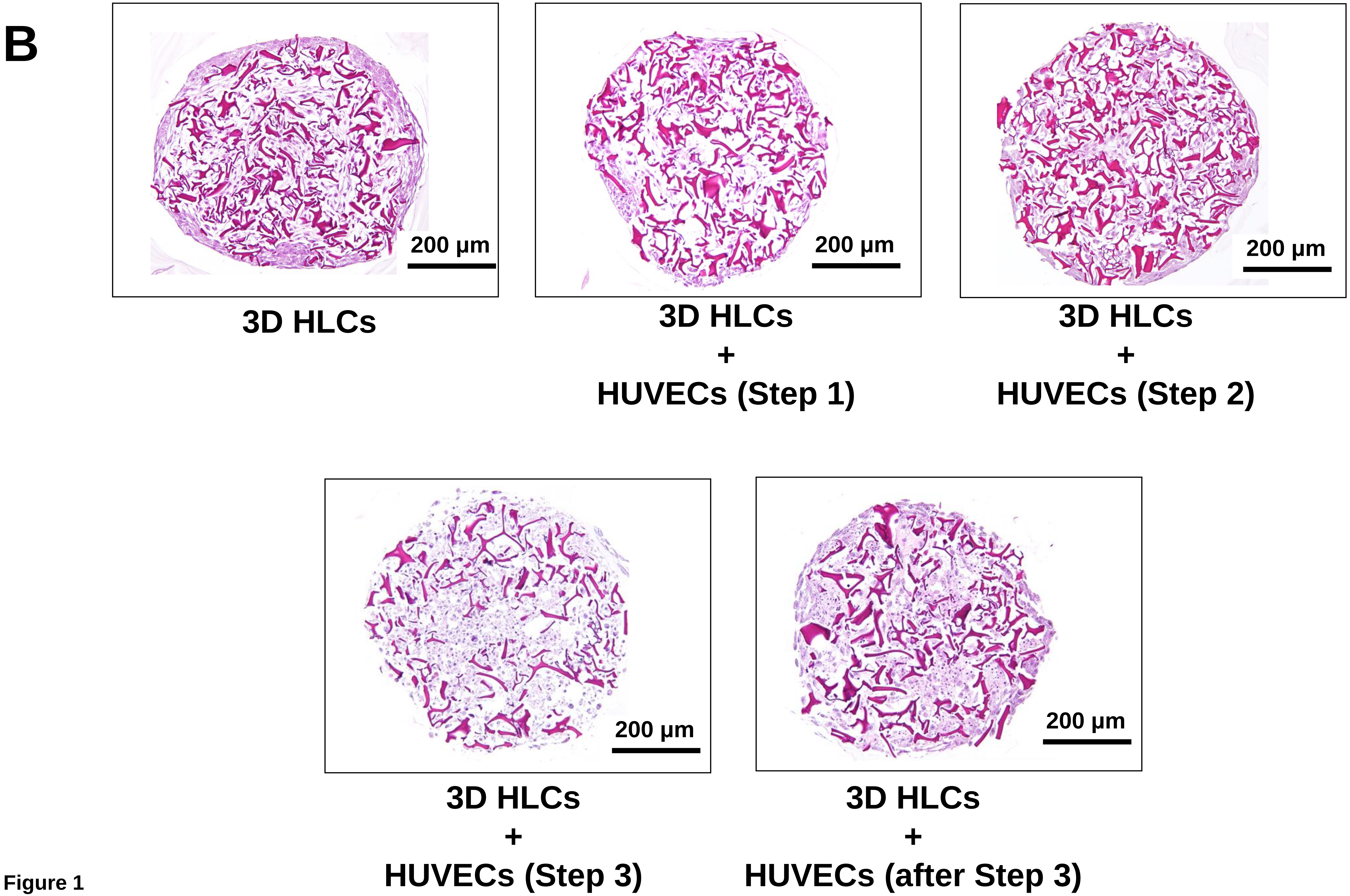
B
200 μm
200 μm
200 μm
3D HLCs
3D HLCs + HUVECs (Step 1)
3D HLCs + HUVECs (Step 2)
200 μm
200 μm
3D HLCs + HUVECs (Step 3)
3D HLCs + HUVECs (after Step 3)

S Figure 1

C

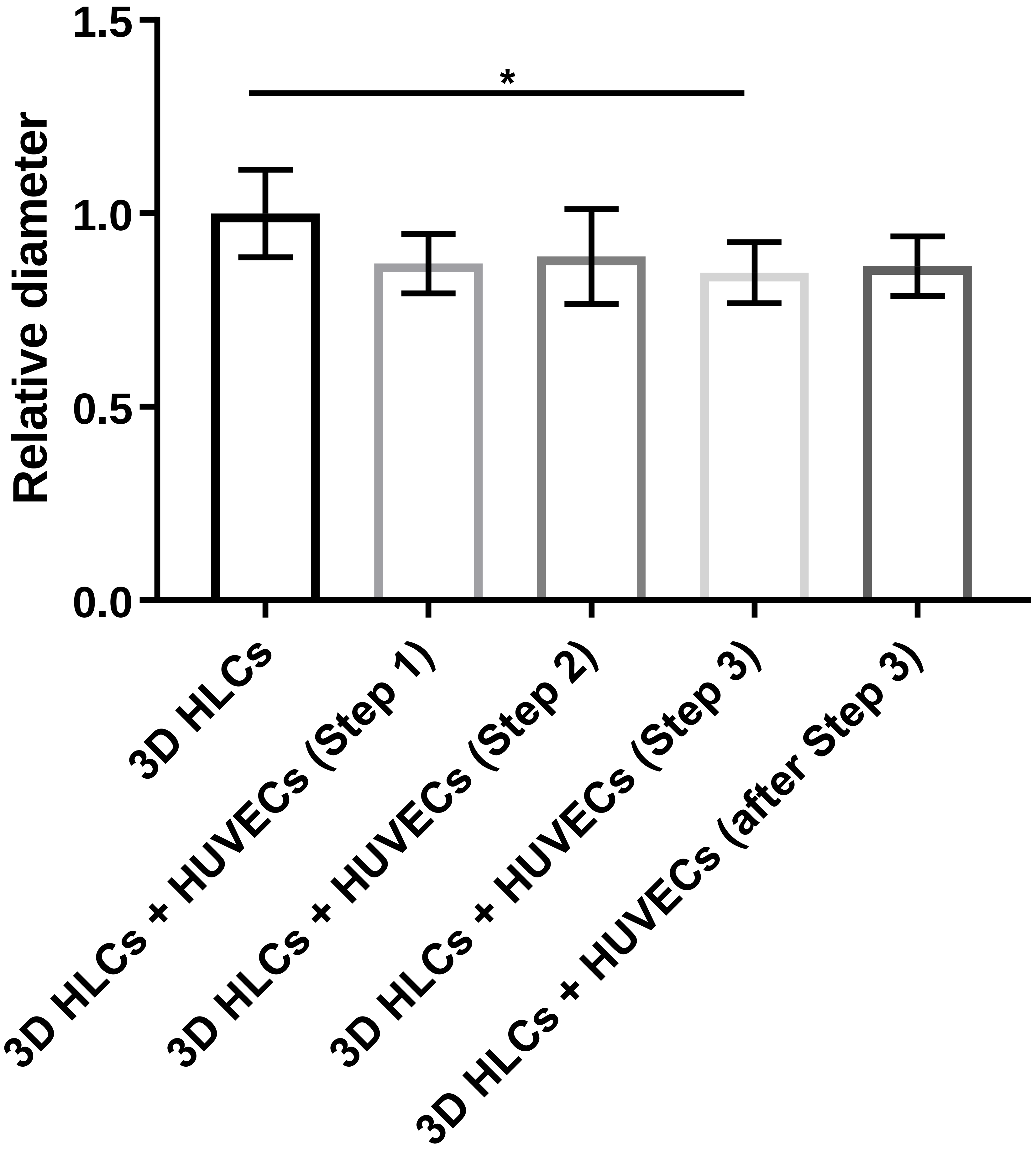

1.5
1.0
0.5
0.0
Relative diameter
*
3D HLCs
3D HLCs + HUVECs (Step 1)
3D HLCs + HUVECs (Step 2)
3D HLCs + HUVECs (Step 3)
3D HLCs + HUVECs (after Step 3)

S Figure 1
